# Laboratory and clinical findings in mouse models of diabetic nephropathy induced with streptozotocin

**DOI:** 10.1186/s12902-023-01504-1

**Published:** 2023-11-22

**Authors:** Aditya Mahardika Wahono, Titut Harnanik, Irma A. Pasaribu, Ronald Pratama Adiwinoto, Yohana Octavianda

**Affiliations:** 1 Medical Student, HangTuah University, Surabaya, Indonesia; 2 Department of Hyperbaric, Faculty of Medicine, Hang Tuah University, Surabaya, Indonesia; 3 Department of Ophtalmology Faculty of Medicine, Hang Tuah University, Surabaya, Indonesia; 4 Department of Community Medicine, Faculty of Medicine, Hang Tuah University, Surabaya, Indonesia; 5 Department of Anatomical Pathology, Faculty of Medicine, Hang Tuah University, Surabaya, Indonesia

**Keywords:** Streptozotozin, Streptozotozin 75 mg/kgBW, Animal model DN

## Abstract

**Background:**

Diabetic nephropathy (DN) represents a microvascular complication of diabetes mellitus (DM). Despite the increasing incidence and prevalence of DN, conservative therapy only reduces risk factors and hemodialysis. This research aimed at finding DN animal model that can be tried to be given an alternative treatment. DN was assessed by evaluating body weight, blood glucose, proteinuria, and kidney histopathology.

**Methods:**

Wistar novergicus male rats were induced with 75 mg of streptozotocin per kg BW to obtain a diabetic nephropathy model. The 18 rats were divided into 2 groups consisting of 9 rats in the negative group (G0) and 9 rats in the positive group (G1). Indicators of body weight, blood glucose levels, urine protein and kidney histopathology determine the incidents of DN animal models.

**Result:**

Rats induced using 75 mg of streptozotocin per kg body weight (BW) indicated weight loss, increased blood glucose, urine protein levels and histopathological features of DN.

**Conclusion:**

Seventy-five mg of streptozotocin per kg BW can induce a diabetic nephropathy animal model in Wistar norvegicus rats.

**Supplementary Information:**

The online version contains supplementary material available at 10.1186/s12902-023-01504-1.

## Introduction

Diabetic nephropathy (DN) is clinically defined as a progressive decline in kidney function caused by chronic hyperglycemia [1]. Functional impairment of DN is manifested by microalbuminuria, kidney hyperfiltration, hyper perfusion and increased capillary permeability to urine macromolecules and proteins with or without chronic kidney insufficiency leading to end-stage kidney disease (ESRD) [2].

The development of ND is associated with many changes in the structure of the kidney compartments. Such early changes as thickening of the glomerular basement membrane (GBM) are seen within 1.5–2 years after the diagnosis of type 1 diabetes mellitus (DM1). In addition, other glomerular changes include the loss of endothelial fenestrations, expansion of the mesangial matrix, and loss of podocytes with thinning of the foot processes. Mesangial expansion is usually detected 5–7 years after the diagnosis of DM type 1. Segmental mesangiolysis is observed in the development of diabetes and is associated with the development of Kimmelstiel Wilson nodules [3].

Thickening of the tubular basement membrane occurs with development of class II glomerular lesions and is prominent in classes III and IV. Interstitial fibrosis and tubular atrophy (IFTA) follow glomerular changes. Vascular lesions are caused by hyalinosis. Hyalinosis usually occurs in the efferent arterioles but may occur in afferent arterioles due to various other alterations. The presence of arteriolar hyalinosis in the kidney could be due to coronary heart disease. Therefore, efferent arteriolar hyalinosis is an essential lesion differentiating diabetic nephropathy from hypertension [4].

Thirty-seven percent of people with type II diabetes mellitus (DM) experience DN in the United States. In the UK, it is evident that Asian people have higher DN incidence than Westerners do. This is because Asian people suffer from type II DM at a relatively younger age so they have a greater chance of DN incidence. Thailand has the prevalence of DN reported at 29.4%, the Philippines at 20.8%, and Hong Kong at 13.1%. In Indonesia the prevalence varies from 2.0% to 39.3% [5]. Among patients requiring dialysis, those with DM had a 22% higher mortality rate at one year and a 15% higher mortality rate at five years than patients without DM [6].

There are two ways to generate a DN model, namely using streptozotocin or alloxan. Ar’Rajab’s research using 240 to 260 g of Spraque Dawley male rats showed that a dose of 30–40 mg/kg resulted in increased glucose levels on days 1 to 7, but it returned to normal on day 10. Whereas at doses of 65–75 mg/kg, despite insulin treatment, plasma glucose levels failed to return to normal [7]. This research aimed at determining the appropriate dose to obtain the DN rat model. Clinical signs of the DN animal model are weight loss, increased blood glucose levels, and urinary protein.

### Ethical statement

The research was conducted in accordance with the Guide for the Care and Use of Laboratory Animals (NIH Publication No. 85–23, revised 1996). The experimental protocol was approved by the Faculty of Medicine, Hang Tuah University (code of ethics No. I/142/UHT. KEPK.03/XI/2022 dated 7 November 2022).

### Housing and animal husbandry

The environment for experimental animals has a temperature of 23 °C and humidity of around 45–65% with a cage sized 120 cm × 70 cm × 60 cm. The rooms, cages and corridors in the experimental animal enclosures must be quiet with drain for the cleaning process. The animal room was acclimated to the animal’s habitat. Rooms were free from chemicals as fumigant. The food given was rat bio, while the drink was boiled tap [8].

### Animal care and monitoring

Physical and environmental checks of the experimental animals were conducted every morning. Food and drinks were replaced 2 times a day in the morning and evening (ad libitum). Feed processing and feed storage must be clean and closed so that it is not easy for insects or other rodents to enter. Drinking water must be checked at all times, especially for any leaks or blockage so that it cannot be accessed by experimental animals. Cages, food and drink bowls must be cleaned regularly to ensure they are hygiene and free from contamination [8].

## Methods

### Research design

This research was carried out through 6 stages, namely stage 1 (arrival and grouping of samples), stage 2 (aclimatization), stage 3 (weighing the rats), stage 4 (initial laboratory examination), stage 5 (injection of 75 mg/kg streptozotozine body weight), stage 6 (final laboratory examination), and stage 7 (termination). The research design used true experimental research. The research used a pre-post test control group design. The experimental unit used Wistar novergicus rats, in which one cage contained five rats. Rat droppings were cleaned every morning.

### Sample

This research used norvegicus wistar rats which were divided into 2 groups. An example of the formula in this Research uses the pre post formula:

$$\mathrm{n}=\frac{{2 ({\mathrm{Z}}_{1}+{\mathrm{Z}}_{2})}^{2} \times2\Theta^2}{\mathrm{d}^2}$$

Description:

n = number of samples per group

Z_1_ = value corresponding to the significance level α

Z_2_ = value corresponding to the strength level β

Θ = standard deviation (baseline std)

d = acceptable difference (pre-post average and post average)

The body weight data were calculated and resulted in pre-post std of (3.87), d value of (37.44), Z1 value of (18), and Z2 value of (2) so that:

$$\mathrm{n}=\frac{{2\left(18+2\right)}^{2}\times2\times 3,87^ {2}}{\mathrm{37,44^2}}$$

$$\mathrm{n}=\frac{2\left(400\right)\times 2\times \mathrm{14,9769}}{\mathrm{1401,7536}}$$

$$\mathrm{n}=\frac{\mathrm{23963,04}}{\mathrm{1401,7536}}$$

$$\mathrm{n }=\mathrm{ 17,09\ }(\mathrm{rounded\ to\ }17)$$

$$\mathrm{Risk\ factor}=\frac{\mathrm{n}\left(1\right)}{1-\mathrm{f}}$$

f = Proportion of sample dropping out, estimated at 10%

$$\mathrm{r^{\prime}}=\frac{17\left(1\right)}{1-\mathrm{0,1}}=\mathrm{18,8\ }\left(\mathrm{rounded\ to\ }18\right)$$

Therefore, the size of sample (population) is 18. Because it consists of 2 groups, then 18:2 = 9. Hence, each group consists of 9 rats.

### Inclusion and exclusion criteria

The research sample used Wistar male rats, approximately 10 months old, in a well condition, and weighing 170–220 g. There were inclusion, exclusion, and drop out criteria in the research sample. The inclusion criteria were male, around 10 weeks old, weighing 170–220 g, and in good physical condition during the research. Rats considered in well condition should have bright eyes, smooth hair, agile movements, good appetite, good body anatomy and no defects, body weight not decreased by more than 10% during the acclimation period. The criterion for exclusion was illness during the acclimation period while the drop out criterion is death during the research process.

### Randomization

This research used streptozotocin-induced Wistar novergicus rats 75 mg/kg body weight divided into 2 groups, namely the negative control group and the positive control group. The negative control group was induced by streptozotocin into diabetic nephropathy animal model. The control group positively induced streptozotocin into diabetic nephropathy animal model.

### Blinding

The researcher did not know the group allocation. The biochemistry laboratory for animal control model, Faculty of Medicine, Hang Tuah University allocated the groups for experimental animals.

### Results of the measurement

In both groups, body weights were measured. Blood sampling (blood glucose measurement) and urine sampling (urine protein measurement) were conducted**.**

### Statistical method

The data collected are in the form of data on body weight, glucose, and urine protein. The data obtained will be separated into 2 groups, namely the negative control group and the positive control group. The data will be analyzed and statistically tested using IBM SPSS to determine the difference between the negative control group and the positive control group. In this research, the dependent variable used a numerical scale for body weight and glucose data, while the nominal scale for urine protein data. The normality of acquired data will be tested using the Shapiro–Wilk test to find out whether the data distribution is normal or not. If the data are normally distributed, it will be continued with the homogeneity test with the Leene test. If the data tested is normal and homogeneous, it will be tested using the T-test parametric test. If the data are not normally distributed, is not homogeneous, and does not meet the parametric requirements, then the data will be tested using the Mann–Whitney U (MWU) test, which is a non-parametric test.

### Experimental animals

**Fig. 1:**
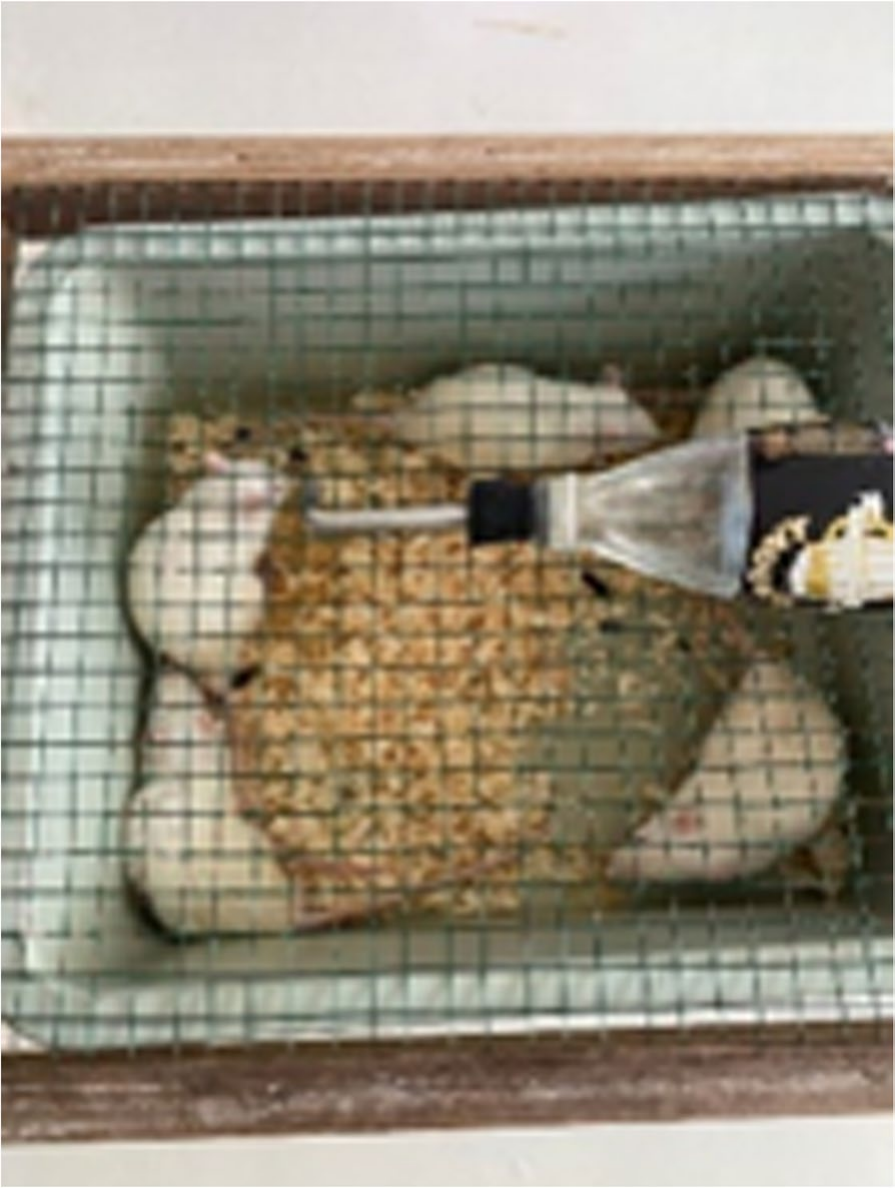
Arrival and grouping of animal model at the Biochemistry Laboratory, Hang Tuah University, in 2022

Diabetic nephropathy was induced in Wistar norvegicus male rats taken from farms in Magetan, East Java, Indonesia. The body weight of the rats used was 170–220 g [7]. The rats were about 10 weeks old. Arriving at the Biochemistry Laboratory, Faculty of Medicine, Hang Tuah University, Surabaya, Indonesia, the rats were acclimated for 7 days (Fig. 1). After 7 days of acclimation, the rats’ body weight was measured before being induced.

After measuring body weight, the rats were induced intraperitoneally by 75 mg streptozotocin per kg BW diluted using citric acid with a pH of 4.5 [7, 9]. On the 7th day after induction, the body weight, blood glucose and urine protein of rats were checked to determine whether there was weight loss, increased blood glucose and urine protein.

### Experimental procedures

#### Body weight measurement

**Fig. 2:**
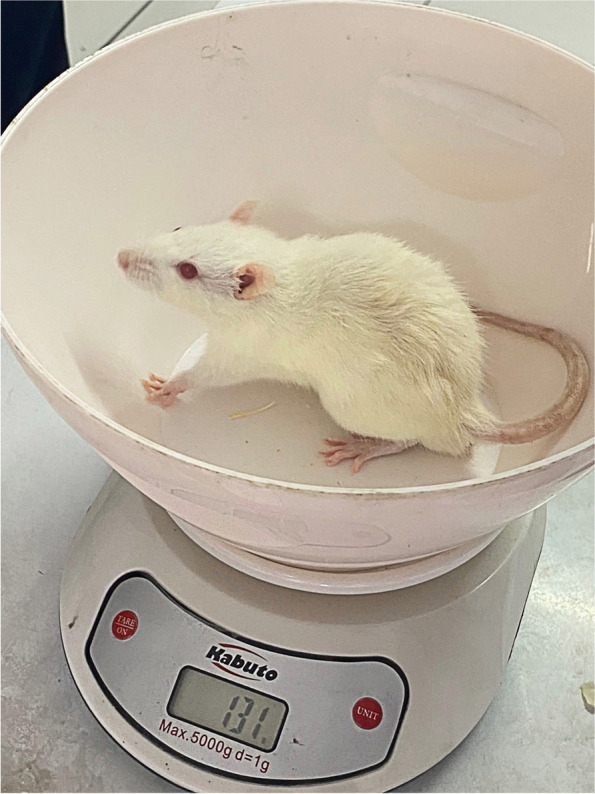
Body weight measurements of animal models at the Biochemistry Laboratory, Hang Tuah University, in 2022

Body weight (BW) was measured twice (7 days after acclimation or before induction and 7 days after induction). BW was measured using a rat scale (Fig. 2). Rats were weighed individually and the results were recorded. When measuring body weight, the scale must be empty or free from anything other than the experimental animal.

#### Blood glucose measurement

In addition to weighing, blood was sampled to check the blood glucose twice, namely after 7 days of acclimation or before induction and 7 days after induction. 0.05 ml of blood was collected in the lateral vein of its tail. Taking a blood specimen with this method must be careful in order to avoid permanent damage to the tail or amputation.

**Fig. 3:**
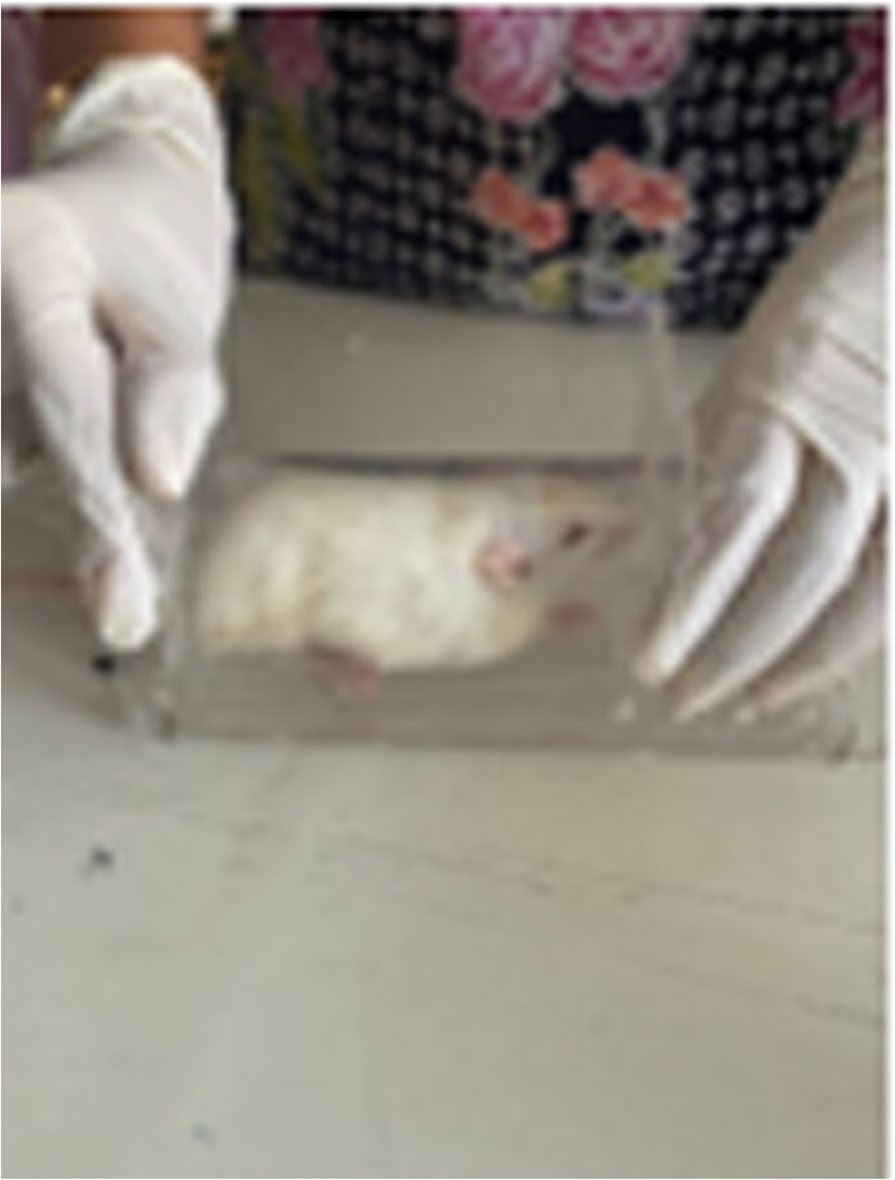
Blood sampling of animal model at the Biochemistry Laboratory, Hang Tuah University, in 2022

Experimental animals were put into shelters (Fig. 3). The rat’s tails were further cut and the blood was taken; It is not allowed to press on the tail as this will lead to damage and contamination of tissue fluids.

#### Urine protein measurement

**Fig. 4:**
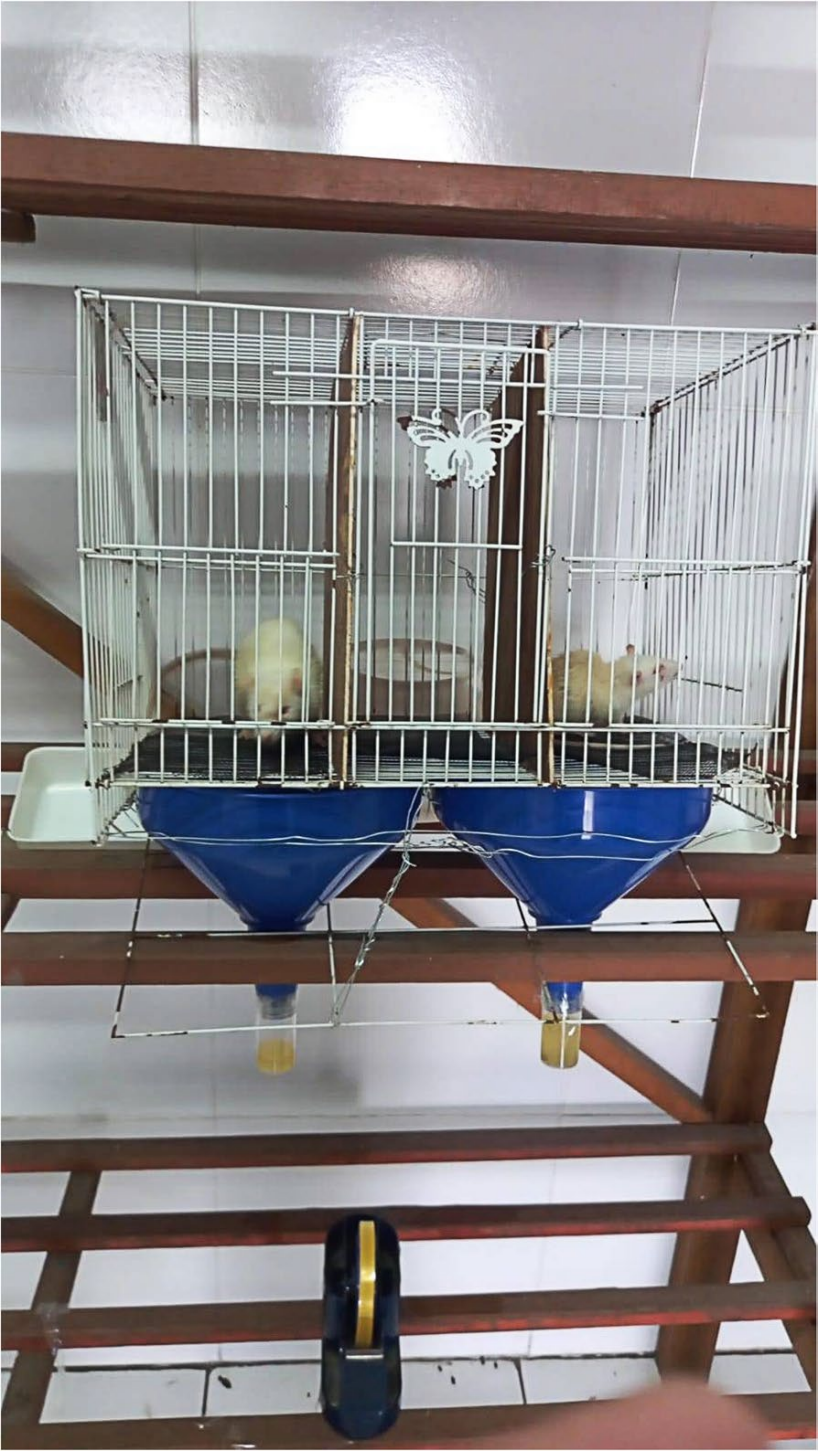
Urine collection of animal model at the Biochemistry Laboratory, Hang Tuah University, in 2022

The rats were housed in special cages for urine collection (Fig. 4). 1 mL of urine must be collected using a urine pot. Rats were fasted in the cage until 1 mL of urine was collected in the urine pot.

Once the urine was full, the rats were transferred to the previous cage, and the urine pot was removed. The urine pot was sent to the biochemistry laboratory, Faculty of Medicine, Hang Tuah University, Surabaya. Urine protein was measured using an immunoturbidimetric test.

#### Collection and preparation of kidney specimens

The kidneys of experimental animals were removed by an abdominal surgery (laparotomy) after anesthesia. Laparotomy started from the lower opening of the abdomen upwards under the diaphragm. Upon completion of the laparotomy, the kidneys were removed. The collected kidneys would be put in 10% formalin [8]. The organs were stained with hematoxylin eosin (HE) and placed in paraffin. The organs in paraffin were read through PAS (periodic acid shift) [4].

#### Anesthesia and euthanization of animals

**Fig. 5:**
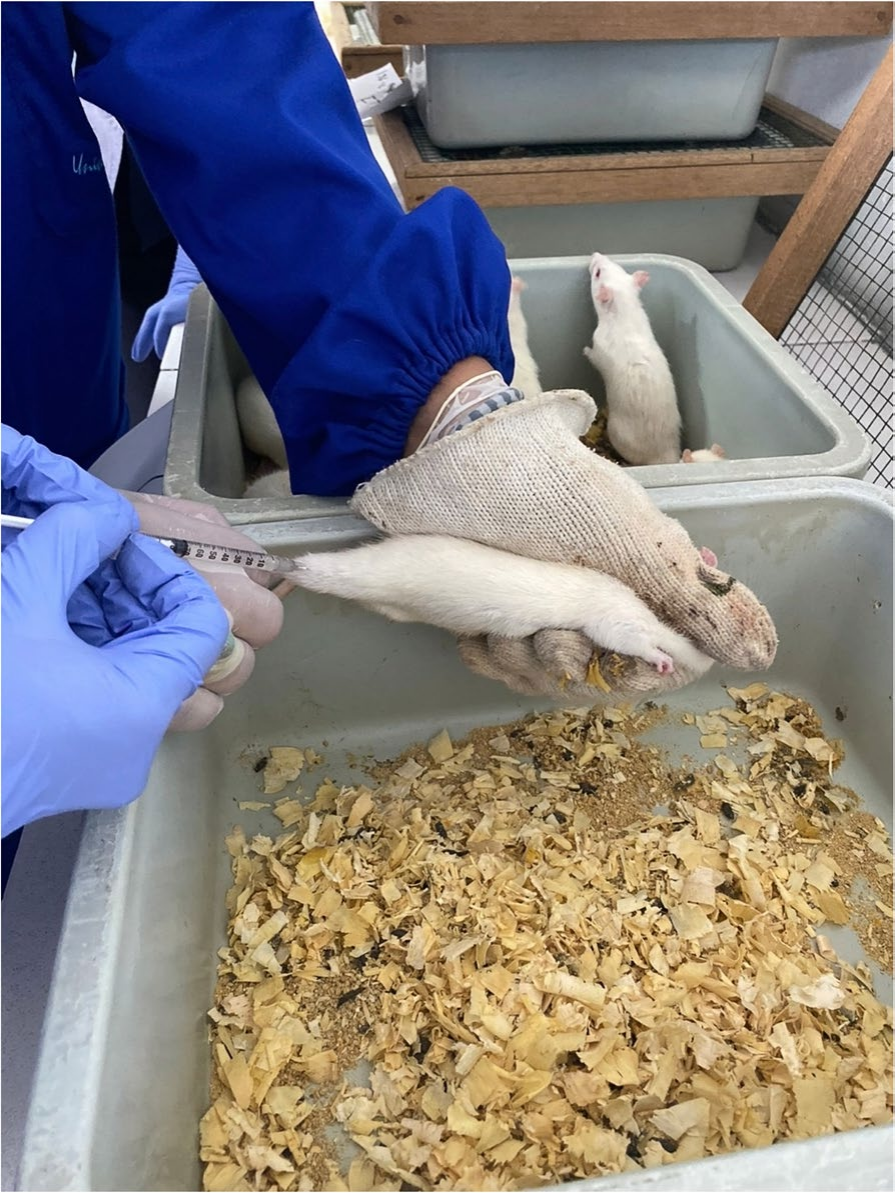
Anesthesia of animal model at the Biochemistry Laboratory, Hang Tuah University, in 2022

Blood was taken intracardiacally. In this method, rats were anesthetized using ketamine 0.5 mg/kg BW (Fig. 5). After that, it took 5 to 10 min until the rats had no pain response. Then the rats underwent laparotomy and blood was taken intracardiacally using a 3 ml syringe. Rats cannot survive after blood was taken intracardiacally [8].

## Results

### Results of weight measurement

The results of weight measurements on day 0 (before STZ-induced) and day 7 after STZ-induced will be displayed in graphical form.

**Fig. 6:**
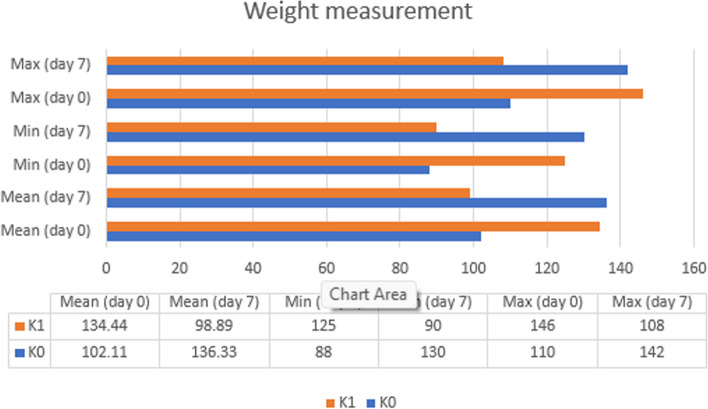
Data of body weight for experimental mice on day-0 and day-7 at the Test Laboratory, Faculty of Medicine, Hang Tuah University in 2023 (*n* = 18). Information: G0 D0: group without STZ induction (negative control group) day 0. G1 D0: group without STZ induction (positive control group) day 0. G0 D7: group without STZ induction (negative control group) day 7. G1 D7: group with STZ induction (positive control group) day 7

In Fig. 6, G0 on day 0 and day 7 showed an increase in body weight (34.22 g). In contrast, after the STZ injection on the G1 day 7, there was a decrease in body weight (-35.55 g) compared with the G1 day 0.

Normal body weight data showed G0 (day 0) of 0.370, G1 (day 0) of 0.269, G0 (day 7) of 0.959, and G1 (day 7) of 0.165. This normality indicated that all data have α > 0.05 which means the data are normal. After conducting the normality test on the data, the homogeneity test must be conducted using the Bonferroni post hoc. On the day 0 the body weight was 0.423, and on day 7 the body weight was 0.014, the homogeneity showed on day 0 the body weight α > 0.05 which means the data are homogeneous; Whereas on the day 7 the weight showed α < 0.05 which means the data are not homogeneous.

On day 0, the body weight data were subject to a parametric test due to normal and homogeneous data; The parametric test used the T-test because it only tested 2 groups. The results of the T-test for body weight on day 0 in groups G0 and G1 found a significant difference (0.000) because α was < 0.05. On the day 7 of body weight data, a nonparametric test was performed because the data were normal but not homogeneous. The nonparametric test used Man Whitney U (MWU) because it only tested 2 groups. The results of the MWU weights on the day 7 in groups G0 and G1 found a significant difference (0.000) because α was < 0.05. In the data on body weight between G1 day 7 and G1 day 0, a nonparametric test was conducted using MWU, the MWU results indicated a significant difference (0.000) between G1 body weight on day 7 and G1 body weight on day 0.

### Results of glucose tests

Glucose test results on day 0 (before STZ-induced) and day 7 after STZ-induced will be displayed in graphical form.

**Fig. 7:**
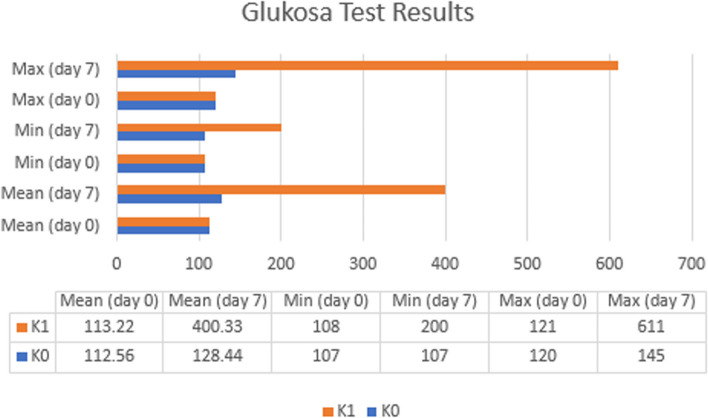
Data of blood glucose for experimental mice on day-0 and day-7 at the Test Laboratory, Faculty of Medicine, Hang Tuah University in 2023 (*n* = 18). Information: G0 D0: group without STZ induction (negative control group) day 0. G1 D0: group without STZ induction (positive control group) day 0. G0 D7: group without STZ induction (negative control group) day 7. G1 D7: group with STZ induction (positive control group) day 7

Figure 7 suggested the results of glucose on day 7 after STZ induced. It was found that on G1 day 7 (400.33) there was an increase in glucose by (287.11) since the day G1 was 0 (113.22). In addition, the glucose ratio at G1 day 7 (400.33) was higher than G0 day 7 (128.44). The maximum and minimum values of G1 on day 0 and day 7 increased, where it had a maximum value (121) and a minimum value (108) on G1 day 0 while it had a maximum value (611) and a minimum value (200) on G1 day 7. The maximum data showed that glucose increased by 490, while the minimum data increased by 92.

Normality test will further be carried out on glucose G0 day 0 of 0.434, G1 day 0 of 0.200, G0 day 7 of 0.573, and G1 day 7 of 0.594. The results of the normality of the data above found that α > 0.05, which means that the data above are normal. Upon the normality test, a homogeneity test was carried out using the Bonferroni post hoc. The result of the homogeneity test in the glucose group on day 0 was 0.768, while on day 7 the glucose group was 0.000. The above results indicated that the glucose group on day 0 had homogeneous data because α > 0.05, while the glucose group on day 7 was not homogeneous because α < 0.05.

The MWU results between G1 day 0 and day 7 showed a significant result of 0.000 (α < 0.05) which explains the significant difference in average. Next, G0 and G1 glucose day 7 was analyzed using MWU because the data were not homogeneous. The results of MWU G0 and G1 glucose day 7 found a significance of 0.000 (α < 0.05) which explains the significant difference in average.

### Results of protein urine tests

The results of urine protein examination on day 0 (before STZ induction) and day 7 after STZ induction will be displayed in tabular form (- and +).

According to Table 1, the urine protein results on G0 and G1 day 0 showed negative (-) because they had not been induced by STZ. After that, urine protein was measured after STZ induction. There was no urine protein on G0 day 7 because it was not induced by STZ, while there was urine protein on G1 day 7 because it was STZ induced.

**Table 1:** Data of protein urine for experimental mice on day-0 and day-7 at the Test Laboratory, Faculty of Medicine, Hang Tuah University in 2023 (*n* = 18)

No	G0 D0	G1 D0	G0 D7	G1 D7
1	Negative	Negative	Negative	Positive
2	Negative	Negative	Negative	Positive
3	Negative	Negative	Negative	Positive
4	Negative	Negative	Negative	Positive
5	Negative	Negative	Negative	Positive
6	Negative	Negative	Negative	Positive
7	Negative	Negative	Negative	Positive
8	Negative	Negative	Negative	Positive
9	Negative	Negative	Negative	Positive
10	Negative	Negative	Negative	Positive
11	Negative	Negative	Negative	Positive
12	Negative	Negative	Negative	Positive
13	Negative	Negative	Negative	Positive
14	Negative	Negative	Negative	Positive
15	Negative	Negative	Negative	Positive
16	Negative	Negative	Negative	Positive
17	Negative	Negative	Negative	Positive
18	Negative	Negative	Negative	Positive

Information:

G0 D0: group without STZ induction (negative control group) day 0

G1 D0: group without STZ induction (positive control group) day 0

G0 D7: group without STZ induction (negative control group) day 7

G1 D7: group with STZ induction (positive control group) day 7

Furthermore, statistical tests were carried out using the MWU test. The results of the MWU G1D0 and G1D7 tests found a very significant difference (0.000), which means that there was a very significant increase in urine protein.

### Results of kidney histopathology

Histopathology was read using a light microscope with 40 × and 100 × magnification. Diabetic nephropathy is assessed according to the International pathological classification of glomerular changes in diabetic kidney disease and divided into 4 classes. Class 1 has GBM thickening and changes. Class 2 is divided into 2a (mild mesangial cell expansion) and 2b (severe mesangial cell expansion). Class 3 has nodular sclerosis and class 4 has advanced diabetic glomerulosclerosis.

In addition to the glomerulus, tubulointerstitial and kidney blood vessels were also assessed. The tubule interstitium is associated with damage to the peritubular interstitial cells that produce erythropoietin. Meanwhile, blood vessels are assessed on the basis of the International classification of interstitial and vascular lesions in diabetic kidney disease, such as interstitial fibrosis and tubular atrophy (IFTA), interstitial inflammation, vascular lesions arteriolar hyalinosis, and the presence of large vessels arteriosclerosis.

**Fig. 8:**
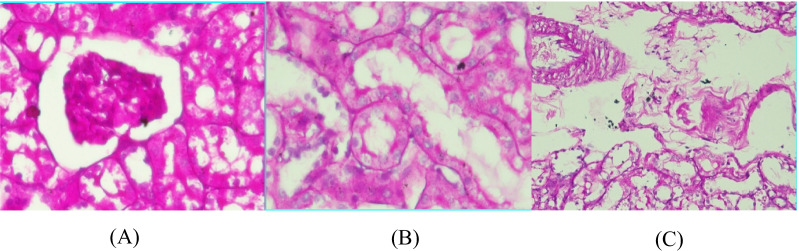
Class 1 diabetic nephropathy histopathology readings; **A** glomeruli, **B** tubule interstitium, **C** blood vessels

Based on the PAS staining, Fig. 8 shows that the glomeruli have mesangial cells within normal limits, slight GBM thickening, no mesangial cells, no increased mesangial matrix forming nodules (Kimmelstiel-Wilson nodules), and no global glomerulosclerosis of > 50%. The tubule interstitium has thickening of the tubular basement membrane, and some atrophic tubules. The blood vessels have an accumulation of hyaline materials in the tunica intima arterioles. In conclusion, the figure is classified as class I diabetic nephropathy.

**Fig. 9:**
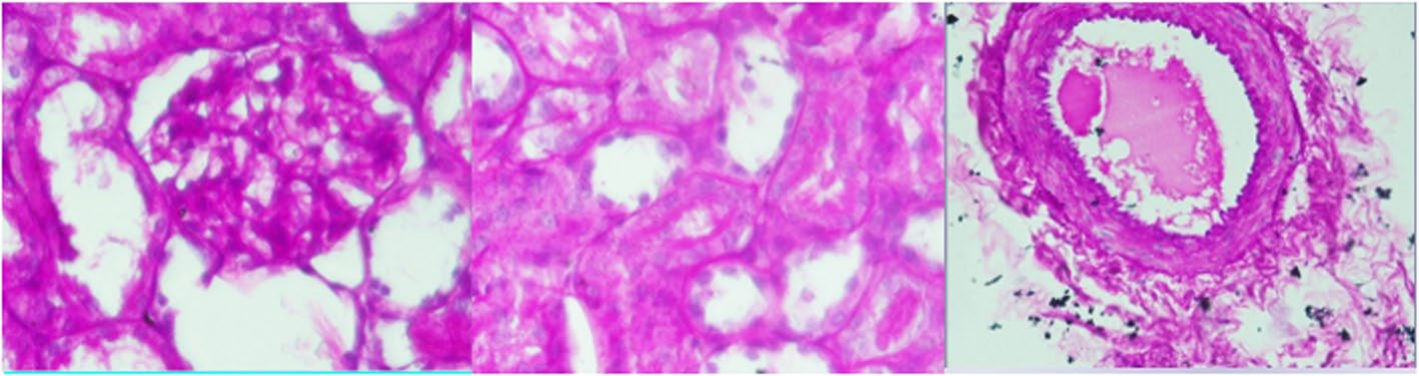
Class IIA diabetic nephropathy anatomic pathology readings; **A** glomeruli, **B** tubulointerstitium, **C** blood vessels

Based on PAS staining, Fig. 9 shows a mild increase in mesangial expansion in glomeruli, slight GBM thickening, no increase in mesangial matrix forming nodules (Kimmelstiel-Wilson nodules), and no global glomerulosclerosis of > 50%. The tubule interstitium has eosinophilic materials, thickened tubular basement membrane, and some atrophic tubules. The blood vessels have an accumulation of hyaline materials in the tunica intima arterioles. In conclusion, the figure is classified as class IIa diabetic nephropathy.

## Discussion

Diabetic nephropathy (DN) is one of the microvascular complications of diabetes mellitus (DM) [10]. Diabetes mellitus (DM) is a chronic metabolic disorder characterized by increased blood glucose levels as a result of insufficient insulin function and is closely related to high mortality rates [11]. In 2011, the incidence of DN due to DM in the US reached 44 million people aged 20–44 years old, 266 million people aged 45–64 years old, and 584 million people aged 65–74 years old [12]. Approximately 20% to 40% of type 2 diabetics with microalbuminuria develop overt nephropathy; and approximately 20% will develop ESRD after the development of overt nephropathy [12]. Hyperglycemia, hypertension, obesity, smoking, race, male, dyslipidemia, age, and genetic factors are the main risk factors for the development and progression of DN [1].

DM sufferers experience insulin deficiency and impaired protein and fat metabolism leading to weight loss. This weight loss results in a reduction in the number of calorie deposits. According to the America Diabetic Association (ADA) the DM disease is characterized by excessive thirst, excessive hunger, frequent urination and weight loss [11].

Weight loss occurs due to a decrease in muscle mass in the body. Weight loss is significantly associated with a decrease in blood glucose. The increase in TNF-α observed in the adipose tissue of obese patients is directly associated with the insulin resistance in obese patients. DM sufferers have a problem with the effect of insulin on the imperfect metabolism of sugar into cells so that blood sugar remains high. This condition can be toxic and cause feelings of weakness and unhealthiness as well as complications and other metabolic disorders [11].

If the body does not get enough energy from sugar, the body will process other substances to be converted into energy such as fat. The use or destruction of fats and proteins leads to weight loss [11].

Diabetes mellitus reduces body weight [11]. It is said that acute and chronic hyperglycemia will reduce body weight. In humans, this weight loss is due to loss of muscle mass. There is no decrease in BW in the G0 group, while G1 group had a significant decrease in BW. This research found that the BW in the G1 group decreased by 26.44 percent.

DM sufferers in a physiological and emotional stress state will trigger hyperglycemia, thereby enhancing glucose production by the liver and disrupting the use of glucose in muscle and fat tissue by opposing the action of insulin [11].

State of stress leads to increased secretion of the epinephrine and cortisol hormones which increase blood glucose levels. Weight gain can be caused by continuous excessive nutritional intake, resulting in excessive fat deposits [11].

Fatty acid deposits in the form of chemical compounds in the form of triacylglycerol found in adipocyte cells can protect the body against the toxic effects of fatty acids. Free fatty acids circulate in the blood vessels throughout the body and cause oxidative stress known as lipotoxicity [11].

The emergence of a lipotoxic effect caused by a number of free fatty acids released by triacylglycerol in an effort to compensate for the destruction of excessive fat deposits will influence adipose and non-adipose tissue, and plays a role in the pathophysiology of diseases in various organs such as liver and pancreas. The consequence of insulin resistance is hyperglycemia, which is compensated for by the synthesis of glucose from the liver (gluconeogenesis), which definitely exacerbates hyperglycemia [11].

The diagnosis of DN is characterized by increased blood glucose levels. Comparison of the average G0 and G1 indicates an increase in average blood glucose. This is theoretically clear that uncontrolled DM will increase the average glucose within a certain time.

Diabetic nephropathy (DN) or diabetic kidney disease is a syndrome characterized by the presence of pathological amounts of urinary albumin excretion, diabetic glomerular lesions, and loss of glomerular filtration rate (GFR) in diabetics. The pathogenesis of diabetic nephropathy involves many factors [13].

In addition to other microvascular complications, the pathogenesis of DN is associated with chronic hyperglycemia. Despite incomplete condition, chronic hyperglycemia leading to DN involves the effects of soluble factors, hemodynamic changes in the renal microcirculation and structural changes in the glomeruli [14].

Functionally, there was initial glomerular hyperfiltration and increased albumin excretion; as nephropathy progresses, there is an increase in proteinuria and a decrease in GFR. Nitric oxide mediates vasodilation of the endothelium, and is formed from L-arginine by endothelial nitric oxide synthase. The rats were knocked out after receiving endothelial nitric oxide synthase due to more severe glomerular lesions and proteinuria. Interstitial macrophage accumulation is strongly correlated with proteinuria, interstitial fibrosis, and reduced GFR [12].

Podocyte slit diaphragm and foot processes are important barriers of glomerular filter, and breakage of the barrier integrity is the leading cause in the progression of proteinuria. Epithelial to mesenchymal transition (EMT) is a response to kidney disease. When EMT occurs, the cytoskeleton of podocytes is rearranged, destroyed their cell junctions and apical-basal polarity, altered their interaction with extracellular matrix, and obtained mesenchymal features, such as invasiveness and elevated motility [15].

Functionally, there was initial glomerular hyperfiltration and increased albumin excretion; as nephropathy progresses, there is an increase in proteinuria and a decrease in GFR. Comparison of G0 and G1 suggests that G1 has proteinuria. This is theoretically clear that DN occurs as due to damage to kidney function causing proteinuria.

Diabetic nephropathy (DN) is characterized by structural and functional changes. Within the glomeruli, there are mesangial expansion, thickening of the basement membrane, and, characteristically, nodular glomerulosclerosis (Kimmelstiel-Wilson nodules). Kidney tissue in diabetic nephropathy is evaluated using renal biopsy. Renal biopsies included haematoxylin and eosin, periodic acid Schiff (PAS), Masson trichrome, and periodic acid-silver methenamine stain for light microscope. The biopsies must contain at least ten glomeruli, excluding incomplete glomeruli along the edges of the biopsy. Immunofluorescence requires the use of antibodies against IgA, IgG, IgM, C3, C1q and kappa and lambda light chains [4].

Histopathologically, there are 5 classes of glomerular injury (class I, IIa, IIb, III, IV) internationally. This glomerular injury evaluates the mesangial glomerular basement, mesangial matrix and glomerulosclerosis). It assesses not only the glomerulus, but also the interstitial tubules and blood vessels.

Class I has a thickened glomerular basement membrane (GBM). Class I biopsies showed no or only mild and non-specific changes by light microscope, and did not meet criteria for classes II to IV (absence of mesangial expansion, no nodular enhancement in the mesangial matrix (Kimmelstiel Wilson’s lesions), and no global glomerulosclerosis more than 50% of glomeruli) [3, 4].

Class II has mild (IIa) to severe (IIb) mesangial expansion. Mesangial expansion is an increase in extracellular material in the mesangium, which leads to an expansion of the gap width outside the two mesangial nuclei by at least two glomerular lobules. The difference between mild and severe mesangial expansion is in terms of the area where the mesangial expansion occurs. Severe mesangial expansion was seen in more than 25% of the total mesangium observed in all biopsies. Mild mesangial expansion is seen in less than 25% of the total mesangium [3, 4].

Class III has nodular sclerosis (Kimmelstiel-Wilson lesions). Class III has at least one Kimmelstiel-Wilson lesion and, on biopsy, the specimen does not have more than 50% global glomerulosclerosis. Kimmelstiel-Wilson lesions appear in type 1 and type 2 DM as focal, lobular, round to oval mesangial lesions with acellular, peripherally rounded, hyaline nuclei/matrix, crescent-shaped mesangial nuclei. Kimmeslstiel-Wilson lesions consist of accumulations of mesangial matrix tissue with collagen fibrils, small lipid particles, and cellular debris [3, 4].

Class IV has advanced diabetic glomerulosclerosis. Class IV has more than 50% global glomerulosclerosis. Glomerulosclerosis of the DN is the end point of a multifactorial mechanism leading to excess accumulation of extracellular matrix proteins, such as collagen types I, III, and IV and fibronectin in the mesangial space which, through the mesangial expansion stages and the Kimmelstiel-Wilson lesion development, results in glomerulosclerosis [3, 4].

After injection of 75 mg of streptozotocin, histopathological examination was made using PAS staining. PAS staining shows changes in the kidneys indicating class I and II. The change in question is damage to the GBM and podocyte cells that have spread.

The pathogenesis of DN is very complex and remains unclear, resulting in poor therapeutic results. Standard therapy, with strict blood sugar and blood pressure failed to halt the progression of DN to ESRD [15] and DN-related mortality [16]. The hypoxic environment in early-stage diabetic nephropathy is exacerbated by manifestations of chronic hyperglycemia, red blood cell abnormalities, oxidative stress, renal sympathetic denervation due to autonomic neuropathy, and diabetes mellitus-induced tubular apoptosis; thus, tubulointerstitial hypoxia in DM may be a critical early event [13].

Although blood glucose and blood pressure levels are well controlled and non-specific measures are also implemented, the development of DN cannot be stopped [1]. Therefore, appropriate DN animal models are needed to try and review the effectiveness of other adjuvant therapies.

This research uses Wistar Rattus norvegicus rats because they weight 170–220 g which will be induced by STZ at a dose of 75 mg/kg. Meanwhile, albino rats only weigh 20–40 g. In addition, these rats only need a single induction of STZ while other rats require 4 inductions of Alloxan. The Wistar strain was chosen because it is easy to find, and is often used for experimental research. Male will not be affected by hormones like female [17].

Streptozotocin (STZ) is a naturally occurring nitrosourea with a molecular weight of 265 and an empirical formula of C14, H27, N5, O12. STZ is widely used to induce diabetes mellitus in experimental animals due to the toxic effects of pancreatic β cells. STZ can be injected as a single injection or as multiple injections at low doses. It is generally induced in male Wistar rats with a body weight of 180–200 g by intraperitoneal administration of diluted 0.01 M citrate buffer, pH 4.5 [9].

Streptozotocin (STZ) prevents insulin release by entering pancreatic cells through the glucose transporter (GLUT 2). STZ further induces activation of polyadenosine diphosphate ribosylation and release of nitric oxide, pancreatic cells are destroyed by necrosis and finally induces insulin dependent diabetes. STZ is selective for pancreatic β cells and results in permanent diabetes in rats, dogs and other species despite relatively resistant rabbits. Its toxicity is through protein Carbamoylation, NDA alkylation, release of free radicals (ROS and RNS), and inhibition of O-GlcNAcse [18].

Based on the report of Jimoe et al., the STZ dose of 25 mg/kg did not result in any changes in serum glucose and immunoreactive insulin or pancreatic insulin levels until 7 days upon induction. Meanwhile, a dose of 100 mg/kg caused cell necrosis, disintegration and phagocytosis of necrotic cells which took place immediately after 3 days of induction by STZ. There was also a remarkable increase in serum glucose within 24 h, and a 98% loss of pancreatic insulin. Doses of 100 mg/kg resulted in the appearance of ketonuria within the first 24 h, and death in most animal models due to loss of insulin determined by exogenous insulin administration. Doses in range of 25 and 100 mg/kg can cause varying severity of diabetes caused by increased doses of STZ, characterized by an increase in serum glucose levels and a progressive decrease in insulin levels at 24 h [7].

Streptozotocin (STZ) is the drug of choice to induce DM in rats due to its ability to cause specific oxidative necrosis caused by pancreatic β cells, hypoinsulinemia, hyperglycemia, and results in multi-organ damage. STZ also develops clinical signs very similar to humans including polyphagia, weight loss (muscle wasting and fat deposits), polyuria, and polydipsia. STZ also causes renal damage and dysfunction because it reduces glomerular filtration rate, Cr clearance, urine output, uremia, micro/macroalbuminuria, glomerulosclerosis, and severe necrosis and apoptosis of renal tubules [19].

This experimental research used STZ because of its stability at any pH, unlike unstable alloxan. In addition, it is easier to generate models using a single induction of STZ compared to alloxan which treats fasted rats with 4 doses. Alloxan is less commonly used than STZ because it has low success rate [20].

The STZ dose used was 75 mg/kg because 25 mg/kg STZ did not produce any changes in serum glucose. Meanwhile, a dose of 100 mg/kg caused cell necrosis, disintegration and phagocytosis of necrotic cells which occurred rapidly after 3 days. There was an uncommon increase in serum glucose within 24 h and a 98% loss of pancreatic insulin, and the appearance of ketonuria within the first 24 h; However, a dose of 100 mg/kg caused death. Most doses in experimental animals are in range of 25 and 100 mg/kg. The severity of diabetes may vary due to increasing doses of STZ, marked increases in serum glucose levels and progressive decreases in insulin levels over 24 h. This severity was reflected in the change in rat weight over 5 weeks at doses of 55 and 65 mg/kg STZ which was failure to gain weight. At doses of 50 to 70 mg/kg, diabetes stabilized. Insulin treatment for 1 week after STZ injection normalized blood glucose in rats that received 55 mg/kg, but failed to give long-term normal plasma glucose in rats receiving 65 to 75 mg/kg [7].

Diabetes mellitus is the name given to several groups of disorders with different etiologies. It is characterized by disorders of carbohydrate, protein, and fat metabolism caused by complete or relative insufficiency of insulin secretion and/or insulin action. This deviation corresponds to acute (fatigue, polyuria, polydipsia, etc.) as well as chronic (retinopathy, neuropathy, nephropathy, peripheral vascular disease, heart failure, etc.) complications of the disease [7]. Those five things (weight loss, increased blood glucose, increased protein composition in urine and DN histopathology) qualify this animal model as the first DN animal model in Indonesia that uses STZ 75 mg/kg BW as mentioned above. Upon the discovery of this DN animal model, it is hoped that any form of adjuvant therapy can be applied.

## Conclusion

Diabetic nephropathy (DN) or diabetic kidney disease is a syndrome characterized by the presence of pathological quantities of urine albumin excretion, diabetic glomerular lesions, and loss of glomerular filtration rate (GFR) in diabetics. The clinical picture of weight loss accompanied by an increase in blood glucose and an increase in the composition of protein in the urine in an animal model of ND is the same as in ND patients. Hopefully this model can be used in finding new alternative therapies for ND patients. The clinical picture of weight loss accompanied by an increase in blood glucose and an increase in the composition of protein in the urine in an animal model of ND is the same as in ND patients. Treatment to delay DN progression involves adequate control of metabolic and hemodynamic abnormalities. Hopefully this model can be used in finding new alternative therapies for ND patients.

### Supplementary Information

**Additional file 1: Table S1.** Laboratory and clinical findings in mouse models of diabetic nephropathy induced with streptozotocin.

## Data Availability

All data generated or analysed during this study are included in this published article in supplementary material.
