# Supplementary material for: Laboratory and clinical findings in mouse models of diabetic nephropathy induced with streptozotocin
Source: BMC Endocr Disord. 2023 Nov 22;23:254. doi: 10.1186/s12902-023-01504-1 (PMC10664383; doi:10.1186/s12902-023-01504-1)
Supplement: Supplementary file 1 — Additional file 1: Table S1. Laboratory and clinical findings in mouse models of diabetic nephropathy induced with streptozotocin. [file 12902_2023_1504_MOESM1_ESM.pdf]

Table S1 Laboratory and Clinical Findings in Mouse Models of Diabetic Nephropathy Induced with Streptozotocin							
Table data of body weight (G0,G1,D0,D&); Glucose (G0,G1,D0, D7), Proteinurine (G0, G1, D0, D7)							
**Number**	**Group**	**Body Weight (D0)**	**Body Weight (D7)**	**Glucose (D 0)**	**Glucose (D 7)**	**Proteinurine (D0)**	**Proteinurine (D7)**
1	G0	88.00	130.00	110	107	Negative	Negative
2	G0	95.00	133.00	113	136	Negative	Negative
3	G0	102.00	135.00	115	141	Negative	Negative
4	G0	105.00	137.00	108	126	Negative	Negative
5	G0	108.00	141.00	107	139	Negative	Negative
6	G0	100.00	134.00	109	145	Negative	Negative
7	G0	107.00	139.00	112	122	Negative	Negative
8	G0	110.00	142.00	119	129	Negative	Negative
9	G0	104.00	136.00	120	111	Negative	Negative
10	G1	125.00	90.00	118	200	Negative	Positive
11	G1	127.00	92.00	108	277	Negative	Positive
12	G1	128.00	93.00	109	205	Negative	Positive
13	G1	128.00	93.00	113	363	Negative	Positive
14	G1	141.00	106.00	112	421	Negative	Positive
15	G1	138.00	102.00	119	551	Negative	Positive
16	G1	141.00	106.00	121	467	Negative	Positive
17	G1	146.00	108.00	111	508	Negative	Positive
18	G1	136.00	100.00	108	611	Negative	Positive
